# Structural analogues of roscovitine rescue the intracellular traffic and the function of ER-retained ABCB4 variants in cell models

**DOI:** 10.1038/s41598-019-43111-y

**Published:** 2019-04-30

**Authors:** Virginie Vauthier, Amel Ben Saad, Jonathan Elie, Nassima Oumata, Anne-Marie Durand-Schneider, Alix Bruneau, Jean-Louis Delaunay, Chantal Housset, Tounsia Aït-Slimane, Laurent Meijer, Thomas Falguières

**Affiliations:** 1 Inserm, Sorbonne Université, Centre de Recherche Saint-Antoine (CRSA), UMR_S 938, Institute of Cardiometabolism and Nutrition (ICAN), F-75012 Paris, France; 2 ManRos Therapeutics, Hôtel de Recherche, Centre de Perharidy, F-29680 Roscoff, France; 3 Assistance Publique - Hôpitaux de Paris, Hôpital Saint-Antoine, Centre de Référence des Maladies Rares - Maladies Inflammatoires des Voies Biliaires & Service d’Hépatologie, F-75012 Paris, France

**Keywords:** Liver diseases, Protein transport, Liver diseases, Protein transport

## Abstract

Adenosine triphosphate binding cassette transporter, subfamily B member 4 (ABCB4) is the transporter of phosphatidylcholine at the canalicular membrane of hepatocytes. ABCB4 deficiency, due to genetic variations, is responsible for progressive familial intrahepatic cholestasis type 3 (PFIC3) and other rare biliary diseases. Roscovitine is a molecule in clinical trial that was shown to correct the F508del variant of cystic fibrosis transmembrane conductance regulator (CFTR), another ABC transporter. In the present study, we hypothesized that roscovitine could act as a corrector of ABCB4 traffic-defective variants. Using HEK and HepG2 cells, we showed that roscovitine corrected the traffic and localisation at the plasma membrane of ABCB4-I541F, a prototypical intracellularly retained variant. However, roscovitine caused cytotoxicity, which urged us to synthesize non-toxic structural analogues. Roscovitine analogues were able to correct the intracellular traffic of ABCB4-I541F in HepG2 cells. Importantly, the phospholipid secretion activity of this variant was substantially rescued by three analogues (MRT2-235, MRT2-237 and MRT2-243) in HEK cells. We showed that these analogues also triggered the rescue of intracellular traffic and function of two other intracellularly retained ABCB4 variants, *i.e*. I490T and L556R. Our results indicate that structural analogues of roscovitine can rescue genetic variations altering the intracellular traffic of ABCB4 and should be considered as therapeutic means for severe biliary diseases caused by this class of variations.

## Introduction

ABCB4, also called MDR3, is a member of the ATP Binding Cassette (ABC) superfamily. Its expression is mainly restricted to the canalicular membrane of hepatocytes where its function is to flop phosphatidylcholine (PC) from the inner leaflet to the outer leaflet of the plasma membrane, thus allowing its secretion into bile (for reviews, see^1,2^). At the molecular level, secreted PC has an essential role since it forms mixed micelles with the other co-secreted hydrophobic bile components, such as bile salts and cholesterol, as first proposed by Admirand and Small^3^. Indeed, impairment of PC secretion into bile leads to the formation of cholesterol crystals and gallstones, as well as loss of protection from the detergent effects of free bile salts on biological membranes of the biliary tree^1,4,5^. These clinical characteristics have been largely reported for patients with rare biliary diseases such as progressive familial intrahepatic cholestasis type 3 (PFIC3), low-phospholipid associated cholelithiasis syndrome or intrahepatic cholestasis of pregnancy^6–8^. These diseases are correlated with genetic variations of the *ABCB4* gene, most of them being missense variations (see^2,9,10^ and references therein, as well as http://abcmutations.hegelab.org/proteinDetails?pid=63). For patients with ABCB4-related biliary diseases, the only pharmacological treatment is the administration of ursodeoxycholic acid (UDCA), a bile acid with low hydrophobicity^11^. Although this therapy is efficient in the milder forms of the diseases^12,13^, UDCA is not – or poorly – efficient in the majority of homozygous or compound heterozygous patients with severe forms of ABCB4-related diseases for whom the only alternative remains liver transplantation^14,15^. Thus pharmacological alternatives are eagerly needed.

The effect of *ABCB4* genetic variations can be classified in five categories, as we recently proposed^9^: no expression of the protein (class I); intracellular retention (class II); impairment of PC secretion activity (class III); defect of protein stability at the canalicular membrane (class IV); no apparent defect (class V). The goal of this classification was to identify targeted pharmacotherapies for the different classes of ABCB4 variants in the frame of personalized medicine^10^. We showed that impaired PC secretion activity of class III ABCB4 variants was corrected by the cystic fibrosis transmembrane conductance regulator (CFTR)/ABCC7 potentiator VX-770/Ivacaftor^®^^16^. Concerning class II variants, cyclosporins have been shown to correct the maturation and the intracellular localisation of endoplasmic reticulum (ER)-retained variants in cell models^9,17–19^. However, cyclosporin A (CsA) inhibits the PC secretion activity of ABCB4^18^, as this is also the case for its close homologue ABCB1/MDR1^20,21^. This clearly indicated that new potential correctors of class II ABCB4 variants had to be sought.

In the present study, we investigated (R)-roscovitine and some newly synthesized analogues as potential correctors for ER-retained ABCB4 variants in Human Embryonic Kidney (HEK) and HepG2 cell models. (R)-roscovitine, also known as Seliciclib or CYC202 and hereafter referred to as roscovitine, is a 2,6,9-trisubstituted purine that was identified as a relatively potent and selective Cyclin-Dependent Kinase (CDK) inhibitor^22–24^. Roscovitine has undergone numerous studies in many indications up to clinical phase trials in various cancers, rheumatoid arthritis, glaucoma and cystic fibrosis (for a review, see^24^). Interestingly, roscovitine has been shown to correct the intracellular localisation and the channel activity of the F508del ER-retained variant of CFTR/ABCC7^25^. Here, we showed that newly synthesized roscovitine analogues correct the maturation, the canalicular expression and more importantly the function of three distinct ER-sequestered ABCB4 variants.

## Results

### Roscovitine rescues the maturation and the canalicular localisation of the I541F ER-retained ABCB4 variant

**Figure 1:**
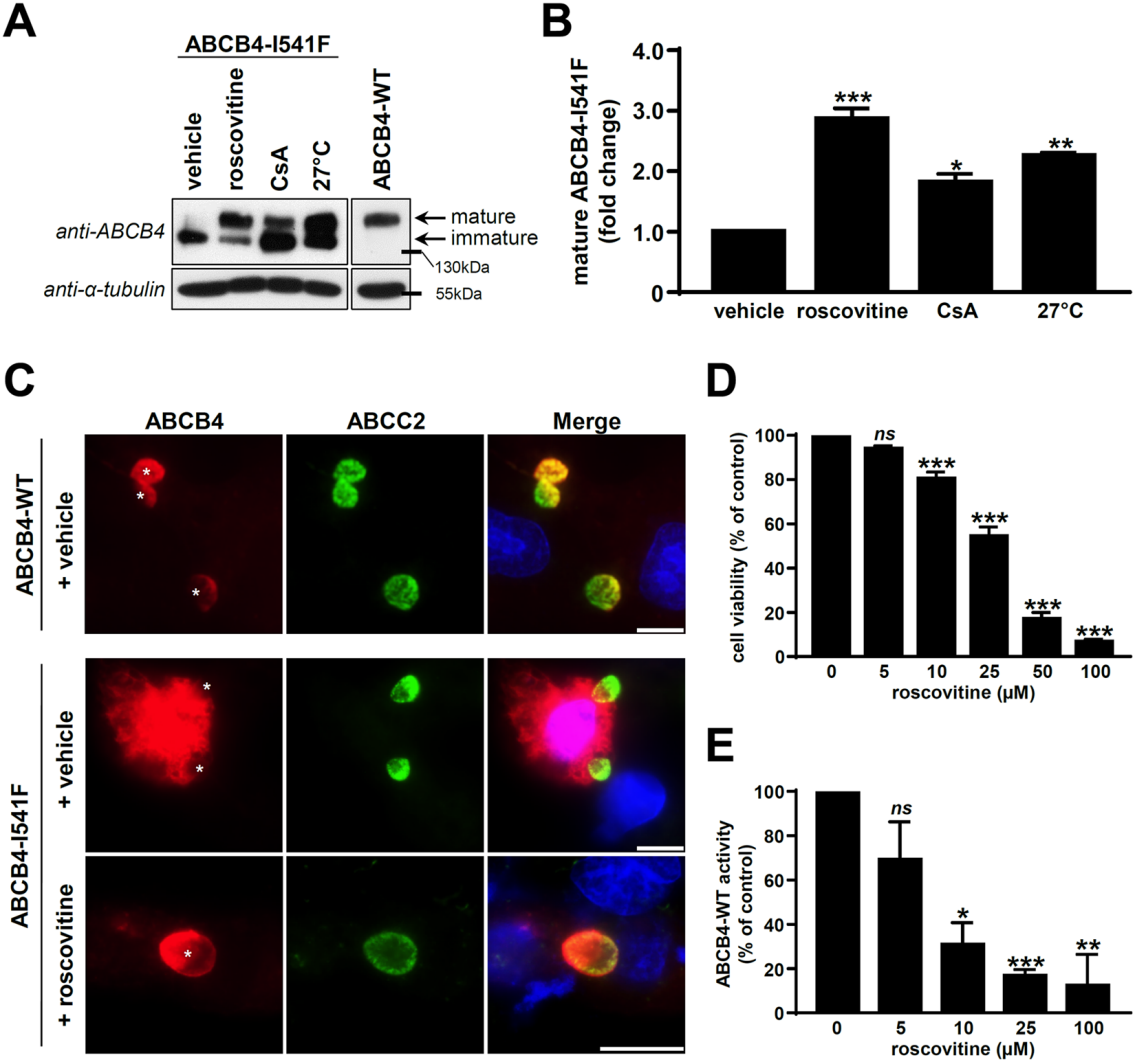
Roscovitine rescues the maturation and the traffic of ABCB4-I541F, but is cytotoxic and inhibitory of ABCB4 activity. (**A**) ABCB4-I541F was transiently expressed in HEK cells. After 16 hours of treatment with vehicle (DMSO), 100 µM roscovitine, 10 µM CsA or cell culture at 27 °C, cell lysates were prepared and analysed by immunoblot using the indicated antibodies. ABCB4-WT expressed under the same conditions is shown as reference. The mature and immature forms of ABCB4 are indicated (arrows). This panel is representative of six independent experiments. Presented data were cropped from full immunoblots shown in Supplementary Fig. S3 and correspond to different exposure times. (**B**) Densitometry analysis of A. The percentage of the mature form of ABCB4 was quantified and then expressed as fold change compared to vehicle-treated cells. Means (±SEM) of six independent experiments are shown. *P < 0.05; **P < 0.01; ***P < 0.001. (**C**) ABCB4-WT or ABCB4-I541F were expressed in HepG2 cells. After 16 hours of treatment with vehicle (DMSO) or 100 µM roscovitine, cells were fixed and permeabilized. After indirect immunofluorescence, ectopically expressed ABCB4 (red) and endogenous ABCC2 (green) were visualized by fluorescence microscopy. Nuclei shown in the merged images were labelled with Hoechst 33342 (blue). Asterisks in the left panels indicate bile canaliculi. This panel is representative of three independent experiments. Bars: 10 µm. (**D**) HEK cells were treated with increasing concentrations of roscovitine during three days. Cell viability was assessed by MTT assay and expressed as a percentage of values for control vehicle-treated cells. Means (±SEM) of four independent experiments performed in triplicate are shown. ***P < 0.001; ns: not significant. (**E**) After transient expression of ABCB4-WT, HEK cells were treated with increasing doses of roscovitine. Then the capacity of these cells to secrete PC was assessed and represented as a percentage of the activity for vehicle-treated control cells (expressing ABCB4-WT) after background subtraction (see *Materials and Methods* for details). Means (±SEM) of at least three independent experiments performed in triplicate for each tested condition are shown. *P < 0.05; **P < 0.01; ***P < 0.001; ns: not significant.

In this study, we used ABCB4-I541F, a prototypical ER-retained variant of ABCB4, first identified in a homozygous PFIC3 patient^7^, and further characterized in our laboratory^17,26^. Indeed, ABCB4-I541F has been shown to be retained in the ER as an immature and high-mannose glycosylated protein^26^, characterized by the absence or low abundance of a mature protein band on immunoblot, compared to the WT protein (Fig. 1A). We have previously demonstrated that the maturation and the localisation at the canalicular membrane of ABCB4-I541F could be partially rescued upon temperature shift of cell culture at 27 °C or treatment with the ABCB1/MDR1 substrate CsA^17,26^. Since roscovitine at 100 µM was described to correct the function of the F508del ER-retained CFTR/ABCC7 variant^25^, we investigated the potential correction of ABCB4-I541F by this molecule. Such treatment led to the correction of the maturation of ABCB4-I541F in HEK cells (Fig. 1A), as shown by the quantification of immunoblots (Fig. 1B). These results were confirmed by indirect immunofluorescence analyses in HepG2 cells, a hepatocellular carcinoma derived cell line forming pseudo-bile canaliculi in cell culture^27^. Indeed, the increased maturation of ABCB4-I541F was associated with its relocalisation at the canalicular membrane after treatment with 100 µM roscovitine in HepG2 cells (Fig. 1C). However, roscovitine displayed important dose-dependent cytotoxicity (Fig. 1D) and inhibition of ABCB4-WT-mediated PC secretion activity in HEK cells (Fig. 1E), whichcompromises its relevance for further investigations.

### Structural analogues of roscovitine rescue the maturation and the canalicular localisation of ABCB4-I541F

**Figure 2:**
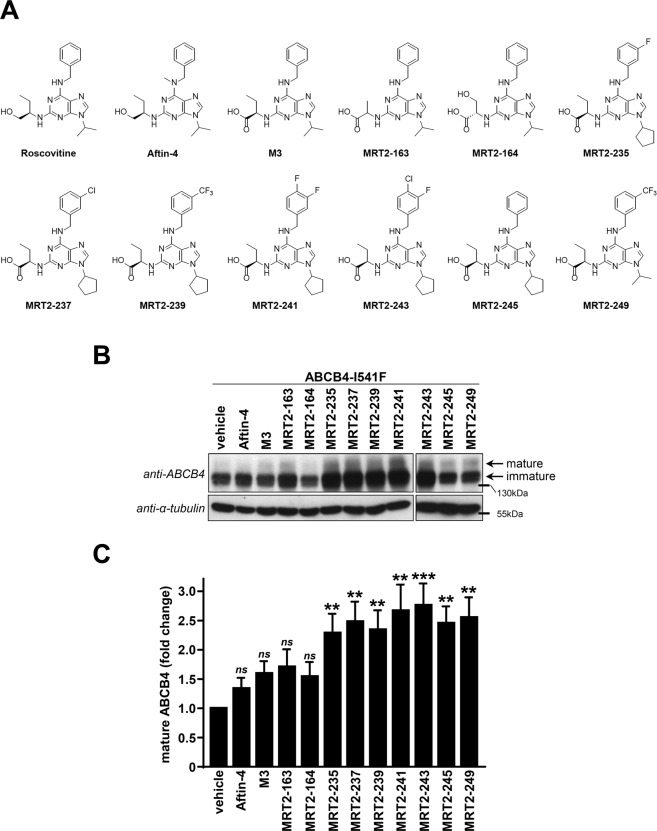
Structural analogues of roscovitine correct the maturation of ABCB4-I541F. (**A**) Structure of roscovitine and its analogues. (**B**) After transient expression of ABCB4-I541F and treatment with 100 µM of the indicated compounds (or DMSO as vehicle), HEK cells were lysed and cell lysates were analysed by immunoblot using the indicated antibodies. The mature and immature forms of ABCB4 are shown. This panel is representative of five independent experiments. Presented data were cropped from full immunoblots shown in Supplementary Fig. S4. (**C**) Densitometry analysis of B. The percentages of the mature form of ABCB4 were quantified and then expressed as fold changes compared to the vehicle-treated condition. Means (±SEM) of five independent experiments are represented. **P < 0.01; ***P < 0.001; ns: not significant.

**Figure 3:**
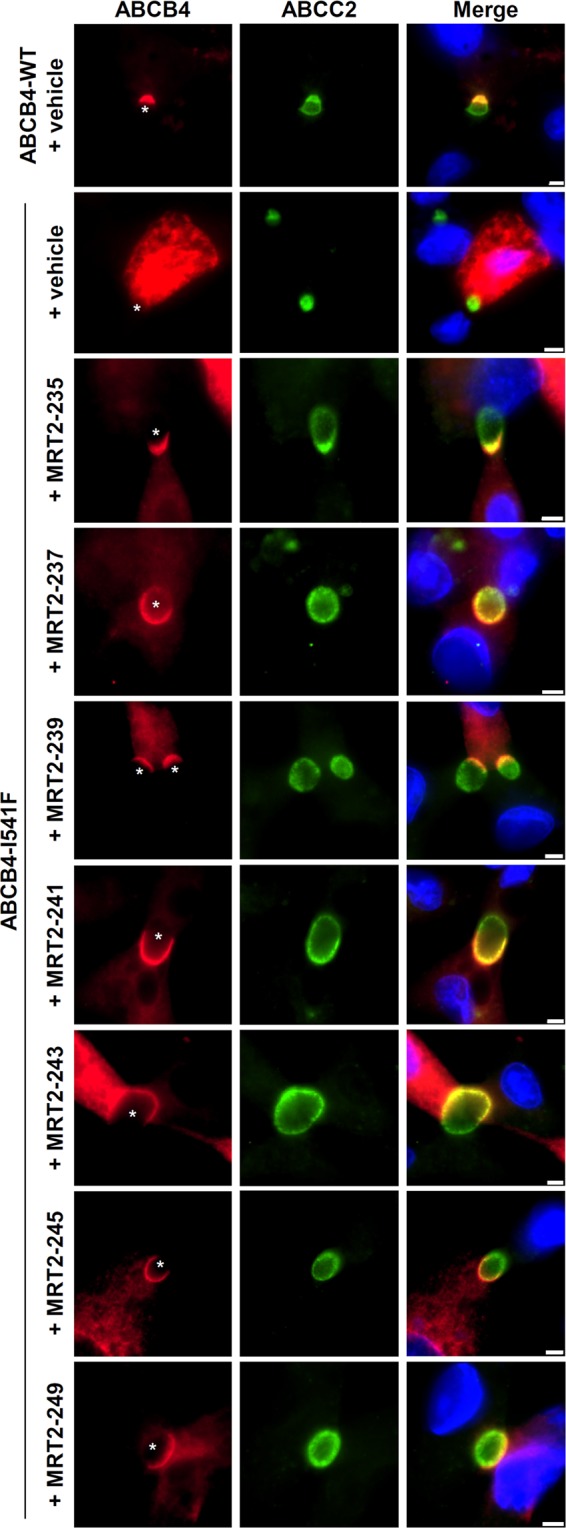
Structural analogues of roscovitine restore canalicular targeting of ABCB4-I541F in HepG2 cells. After transient expression of ABCB4-WT or ABCB4-I541F and 16 hours of treatment with the vehicle (DMSO) as control or 100 µM of the indicated compounds, HepG2 cells were fixed and processed for indirect immunofluorescence and fluorescence microscopy to visualize ABCB4 (red, left panels) and ABCC2 (green, middle panels). Nuclei shown in the merge images were labelled with Hoechst 33342 (blue, right panels). Asterisks in the left panels indicate bile canaliculi. This figure is representative of at least three independent experiments per condition. Bars: 5 µm.

The cytotoxic effect induced by roscovitine might be explained by its CDK inhibition activity^22^. We thus synthesized structural analogues (Fig. 2A) potentially devoid of this feature. Aftin-4 is a methyl-roscovitine with reduced kinase inhibition activity^28,29^, and M3 is the main hepatic metabolite of roscovitine. All other products are carboxylated analogues displaying much reduced kinase inhibition compared to the parent compound, roscovitine (Table 1). This indicates that carboxylation at the hydroxyl level and/or methylation at position N6 (Fig. 2A and Supplementary Fig. S1) strongly reduce CDK inhibition activity of these molecules. As expected, all roscovitine analogues with decreased CDK inhibition activity were less cytotoxic (Table 1 and Supplementary Fig. S1). Interestingly, in HEK cells, some of these analogues (MRT2-235, -237, -239, -241, -243, -245 and -249) were able to significantly increase the expression of a mature form of ABCB4-I541F while other analogues (Aftin-4, M3, MRT2-163 and MRT2-164) were not (Fig. 2B). The quantification of these results indicated a ~2.5 fold increase of expression of the mature form of ABCB4-I541F after treatment with 100 µM of the most potent roscovitine analogues (Fig. 2C). We then analysed the efficacy of the most potent analogues to correct the canalicular targeting of ABCB4-I541F in HepG2 cells. After 16 hours of treatment with 100 µM of these compounds, we observed partial relocalisation of ABCB4-I541F at bile canaliculi, as shown by its partial co-localisation with ABCC2 (Fig. 3). Altogether, these results demonstrate that the maturation and the canalicular localisation of ABCB4-I541F can be rescued by new structural analogues of roscovitine.

**Table 1:** IC_50_ of roscovitine and its analogues towards various protein kinases (µM).

Kinases	CDK2/A^a^	CDK5/p25^a^	CDK9/T^a^	CK1	CLK1	DYRK1A	GSK3
Roscovitine	0.080	0.210	0.533	4.3	2.9	3.33	>10
M3	9.0	20	>33	>33	>33	19	>10
MRT2-163	6.0	20	>33	20	>33	>10	>10
MRT2-164	2.2	10	17	28	>33	>10	>10
MRT2-235	5.9	8.3	7	7	11	21	>10
MRT2-237	3.4	7.7	9	3.33	8.5	13	>10
MRT2-239	8.0	11	20	4	9	21	>10
MRT2-241	5.1	17	8	11	13	20	>10
MRT2-243	3.0	6.3	5	6.1	20	12	>10
MRT2-245	6.3	15	28	17	13	21	>10
MRT2-249	9.0	27	>33	5.0	23	9	>10

Abbreviations: CDK, cyclin-dependent kinase; CK1, casein kinase 1; CLK1, Cdc2-like kinase 1 (CLK1); DYRK1A, dual specificity tyrosine phosphorylation regulated kinase 1A; GSK3, glycogen synthase kinase 3B.

^a^The activators/regulators of CDKs are indicated after the slashes: A, cyclin A; p25; T, cyclin T.

### Structural analogues of roscovitine are less cytotoxic and less inhibitory of ABCB4 activity than roscovitine

**Figure 4:**
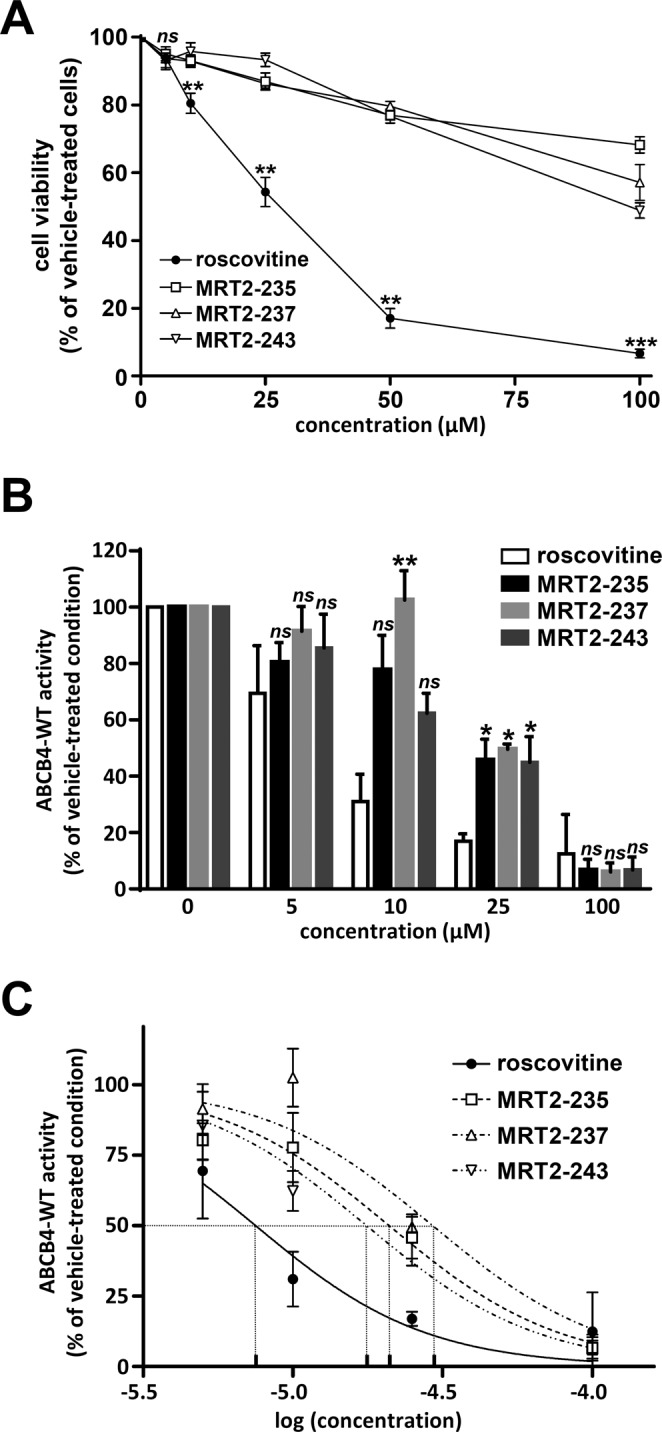
Roscovitine analogues are less cytotoxic and less inhibitory of ABCB4 activity than roscovitine. **(A**) After treatment with increasing concentrations of the indicated compounds during three days, cell viability was determined and expressed as in Fig. 1D. Means (±SEM) of at least four independent experiments per condition performed in triplicate are shown. Statistics indicate comparisons between roscovitine treatment and the other conditions for each tested concentration: **P < 0.01; ***P < 0.001; ns: not significant. (**B**) After transient expression of ABCB4-WT, HEK cells were treated with increasing doses of roscovitine or its analogues as indicated. Then the capacity of these cells to secrete PC was assessed and expressed as in Fig. 1E. Means (±SEM) of at least three independent experiments performed in triplicate for each tested condition are shown. Statistics indicate comparisons between treatments with roscovitine and its analogues for each tested concentration: *P < 0.05; **P < 0.01; ns: not significant. (**C**) Results shown in B are represented as functions of log[concentration] for roscovitine and its analogues.

To pursue this study, we decided to focus on three roscovitine analogues (MRT2-235, MRT2-237 and MRT2-243) which displayed significant rescue of the maturation and partial relocalisation of ABCB4-I541F at bile canaliculi (Figs 2B,C and 3). At concentrations lower than 100 µM, these analogues were much less cytotoxic than roscovitine in HEK cells (Fig. 4A). Indeed, the cell viability was still higher than 80% at 25 µM of the analogues while it was reduced to less than 60% at the same concentration for roscovitine (Fig. 4A). Furthermore, measurement of ABCB4-WT-mediated PC secretion in HEK cells indicated that the three roscovitine analogues were less inhibitory than the original molecule when used at 10 µM and 25 µM (Fig. 4B). The IC_50_ on ABCB4-WT activity was 7.5 µM for roscovitine while it was 2.5 to 4.0 fold higher for its analogues (Fig. 4C and Table 2). It is also important to notice that treatment with roscovitine and these three analogues at 10, 25 and 100 µM do not alter the plasma membrane localisation of ABCB4-WT in HEK cells (Supplementary Fig. S2). Thus, inhibition of ABCB4-mediated PC secretion by roscovitine and its analogues may be due to a direct effect of these compounds on ABCB4 function. Altogether, these results indicate that roscovitine analogues might be interesting ABCB4 correctors with low cytotoxicity and low inhibition activity of ABCB4.

**Table 2:** IC_50_ of roscovitine and its analogues on the PC secretion activity of ABCB4-WT.

Compounds	Roscovitine	MRT2-235	MRT2-237	MRT2-243
log IC_50_	−5.124 ± 0.078	−4.678 ± 0.086	−4.533 ± 0.092	−4.754 ± 0.084
IC50 (µM) *(min-max)*	7.51 *(5.49–10.28)*	20.97 *(14.87–29.62)*	29.31 *(20.30–42.32)*	17.63 *(12.60–24.64)*

IC_50_ were calculated from dose-response curves shown in Fig. 4C.

### Roscovitine analogues rescue the maturation, the localisation and the activity of ABCB4-I541F

**Figure 5:**
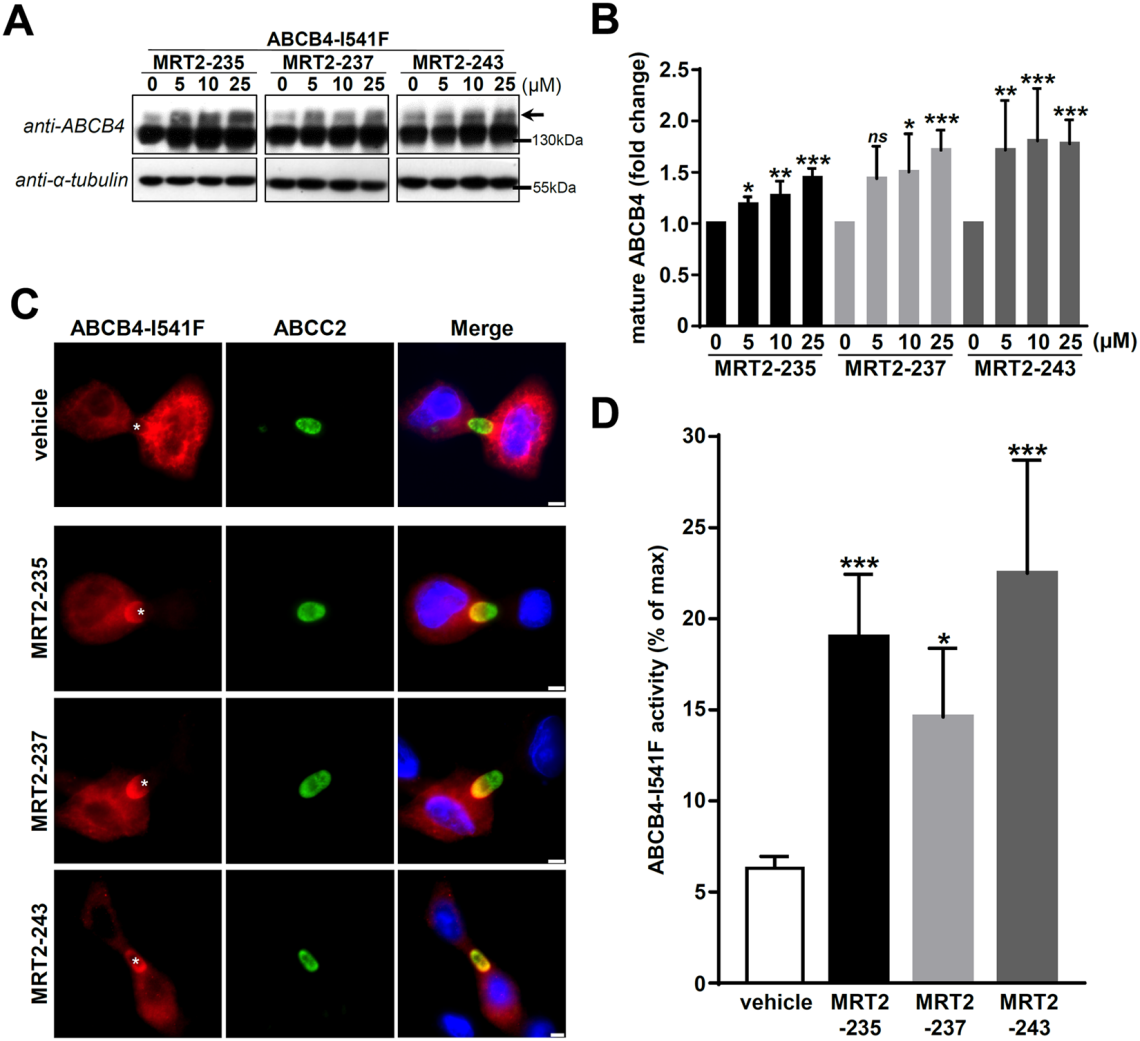
Roscovitine analogues rescue the maturation, the plasma membrane targeting and the PC secretion activity of ABCB4-I541F. (**A**) After transient expression of ABCB4-I541F in HEK cells and treatment with 0 (vehicle), 5, 10 or 25 µM of the indicated roscovitine analogues, cell lysates were prepared and analysed as in Fig. 2B. The arrow indicates the mature form of ABCB4. These immunoblots are representative of at least five independent experiments for each condition. Presented data were cropped from full immunoblots shown in Supplementary Fig. S5. (**B**) Densitometry analysis of A. The percentages of the mature form of ABCB4 were quantified and then expressed as fold changes compared to the vehicle-treated condition (0 µM) for each tested compound. Means (±SEM) of at least five independent experiments per roscovitine analogue are represented. *P < 0.05; **P < 0.01; ***P < 0.001; ns: not significant. (**C**) After transient expression of ABCB4-I541F, HepG2 cells were treated during 16 hours with 25 µM of the indicated roscovitine analogues. ABCB4 (red, left column) and ABCC2 (green, middle column) were immunolocalised as in Fig. 3. Nuclei (blue) are shown in the merge panels (right column). Asterisks in the left panels indicate bile canaliculi. This panel is representative of three independent experiments. Bars: 5 µm. (**D**) HEK cells expressing ABCB4-I541F were treated with 25 µM of roscovitine analogues, and ABCB4-mediated PC secretion was measured and represented as in Fig. 1E, the maximal activity being determined for cells expressing ABCB4-WT. Means (±SEM) of at least five independent experiments performed in triplicate for each tested condition are shown. *P < 0.05; ***P < 0.001.

The inhibition of ABCB4 activity induced by MRT2-235, MRT2-237 and MRT2-243 when used at 100 µM led us to investigate these analogues at lower concentrations. After treatment with 5, 10 and 25 µM of the three analogues, we observed a dose-dependent correction of ABCB4-I541F maturation in HEK cells (Fig. 5A; quantification in Fig. 5B). Treatment with 25 µM of these analogues triggered partial relocalisation of ABCB4-I541F at bile canaliculi in HepG2 cells (Fig. 5C). Finally, we measured the PC secretion activity of ABCB4-I541F in HEK cells after treatment with or without roscovitine analogues at 25 µM. Importantly, we noticed a marked correction of ABCB4-I541F activity from 7.1 ± 0.8% of residual activity with control treatment (vehicle) to 19.0 ± 3.4%, 13.0 ± 3.5% and 22.5 ± 6.2% after treatment with 25 µM of MRT2-235, MRT2-237 and MRT2-243, respectively (Fig. 5D). Altogether, our results demonstrate that the three selected roscovitine analogues are able to retrieve the maturation and the canalicular localisation of the ER-retained ABCB4-I541F variant and to significantly correct its PC secretion activity.

### Roscovitine analogues rescue the function of two other ER-retained ABCB4 variants

**Figure 6:**
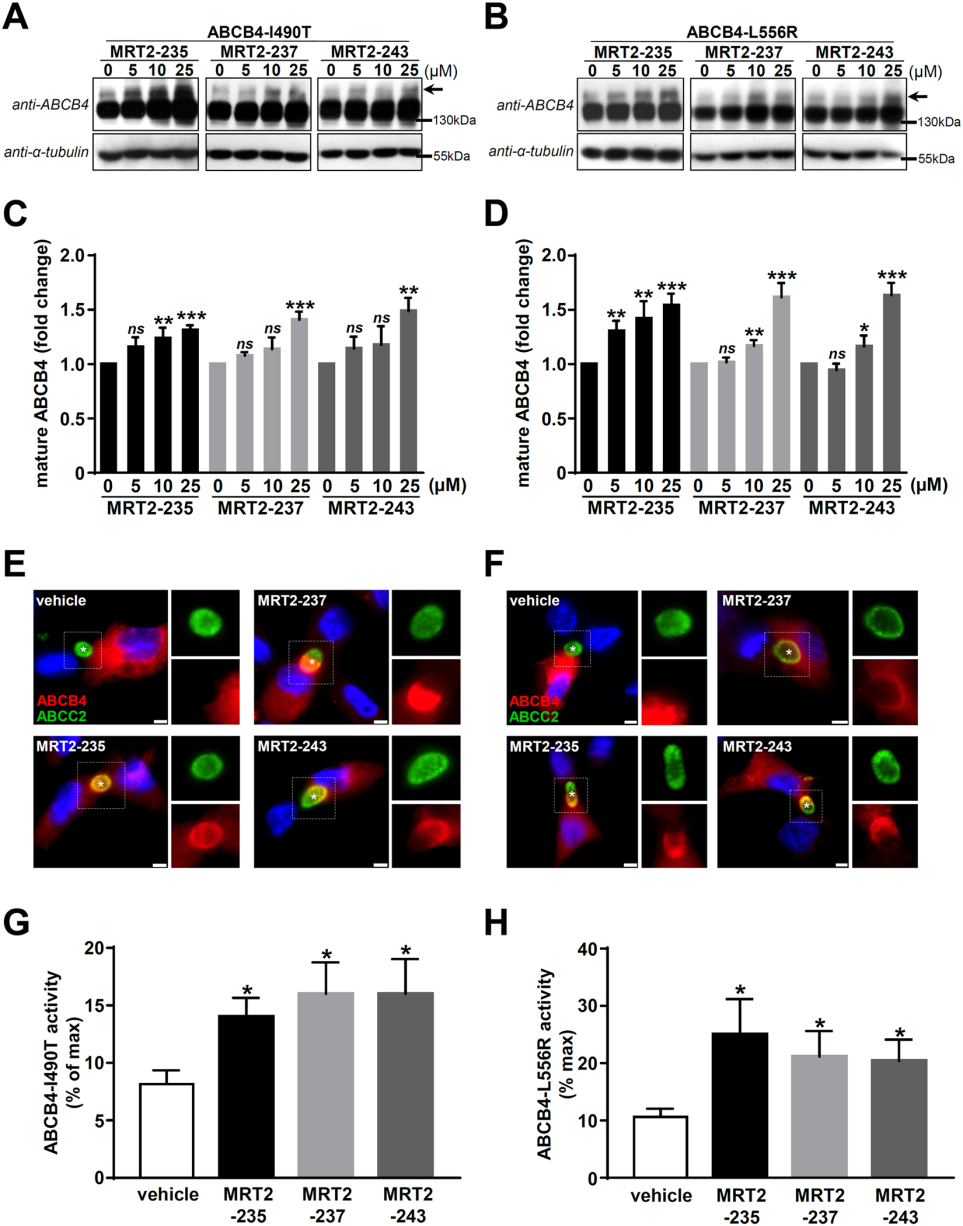
Roscovitine analogues are functional correctors of other ER-retained ABCB4 variants. **(A**,**B**) After treatment with 0 (vehicle), 5, 10 or 25 µM of the indicated roscovitine analogues, the maturation of ABCB4-I490T (**A**) and ABCB4-L556R (**B**) missense variants expressed in HEK cells was assessed by immunoblot as in Fig. 5A. These panels are representative of at least four independent experiments for each condition. Presented data were cropped from full immunoblots shown in Supplementary Fig. S6. (**C**,**D**) Densitometry analyses of A and B, respectively, as performed in Fig. 5B. Means (±SEM) of at least four independent experiments per condition are represented. *P < 0.05; **P < 0.01; ***P < 0.001; ns: not significant. (**E**,**F**) After treatment without (vehicle) or with 25 µM of the indicated roscovitine analogues, HepG2 cells expressing ABCB4-I490T (**E**) or ABCB4-L556R (**F**) were processed for indirect immunofluorescence, as described in Fig. 5C. White dotted squares indicate magnified areas in the individual frames shown on the right of each merged picture. Asterisks indicate bile canaliculi. Each panel is representative of three independent experiments. Bars: 5 µm. (**G**,**H**) ABCB4-mediated PC secretion of HEK cells expressing ABCB4-I490T (**G**) or ABCB4-L556R (**H**) and treated with 25 µM of the indicated roscovitine analogues was analysed as in Fig. 5D. Means (±SEM) of at least four independent experiments performed in triplicate for each tested condition are shown. *P < 0.05.

To extend this study, we analysed the effect of roscovitine analogues on two other ER-retained missense ABCB4 variants, I490T and L556R, identified in patients with liver cancer^30^ or PFIC3^7^. Without treatment, these variants were mainly detected as immature proteins by immunoblot in HEK cells (Fig. 6A,B). However, after treatment with 5 to 25 µM of the three roscovitine analogues, we observed a dose-dependent rescue of the maturation of these two variants (Fig. 6A,B), as confirmed by the quantification of these experiments (Fig. 6C,D). These results were further validated by indirect immunofluorescence assays in HepG2 cells: both I490T and L556R variants were partially relocalised at bile canaliculi upon treatment with 25 µM of MRT2-235, MRT2-237 or MRT2-243 (Fig. 6E,F). As expected, the PC secretion activity of these two ER-retained variants in HEK cells was strongly impaired in vehicle-treated cells (Fig. 6G,H). However, after treatment with 25 µM of the three roscovitine analogues, we observed a significant increase of the residual activity of these variants (Fig. 6G,H). These results provide evidence that roscovitine analogues are able to rescue the localisation, the maturation and the PC secretion activity of several ER-retained ABCB4 variants.

## Discussion

PFICs and hereditary cholestatic diseases in general represent a considerable fraction of indications for liver transplantation^31,32^. In the absence of surgery and efficient pharmacological treatment able to cure patients, or at least to delay liver transplantation, the life expectancy is low^33^. Roscovitine has been shown to partially rescue intracellular traffic and chloride channel activity of CFTR-F508del^25^. Thus, in this work, we investigated the possibility to repurpose this molecule in the frame of ABCB4-related diseases. While we observed that roscovitine was able to correct maturation and localisation of the ER-retained ABCB4-I541F variant in cell models, its important cytotoxicity and inhibition of ABCB4-WT function ruled out its use for further analyses. We then synthesized structural analogues of roscovitine, devoid of intrinsic CDK inhibition capacity (Table 1). Interestingly, three of these analogues (MRT2-235, MRT2-237 and MRT2-243) were less cytotoxic and less inhibitory of ABCB4-WT activity than the original molecule, and allowed partial rescue of the maturation and the localisation of distinct ER-retained ABCB4 variants. More importantly, these corrections were associated with a significant rescue of PC secretion for these variants. Thus, roscovitine analogues may be considered as good candidates in preclinical studies.

We and others have previously shown that cyclosporins, essentially CsA, are able to rescue the maturation and the cell surface localisation of ABCB4 defective variants^9,17–19^. However, until now, attempts to rescue functional ABCB4 variants using substrates and/or inhibitors of ABCB1 such as CsA, FK506/Tacrolimus or Tariquidar were not successful (^18^; our unpublished data), strengthening the requirement for developing new targeted pharmacotherapies. Here, we provide evidence that roscovitine analogues are efficient correctors of the intracellular traffic as well as the function of ER-retained ABCB4 variants. The chemical chaperone 4-phenylbutyrate, already used in clinics^34,35^, has been shown to rescue other ER-retained ABCB4 variants^36^, as well as defective variants of different ABC transporters (^10^ and references therein). However, for patients who do not respond or comply to this therapy, or develop major side effects^37,38^, roscovitine analogues may constitute good alternatives in the frame of personalized medicine.

Roscovitine has been shown by direct co-cristallisation approaches to bind to CDK2 and CDK5 in their catalytic site, and to directly compete with ATP^39,40^. We can speculate that ABCB1 substrates, such as CsA, correct ABCB4 defective variants but inhibit its function by similar mechanisms. In the present study, while roscovitine analogues allowed a better folding of ER-retained ABCB4 variants and thus their cell surface relocalisation, these molecules could impair the PC secretion activity by additional molecular mechanisms. Thus, roscovitine analogues seem to be dual molecules with two distinct functions: one allowing a better folding and traffic of ER-retained ABCB4 variants and the other interfering with the PC secretion activity of the transporter.

Roscovitine analogues may correct ABCB4 function through different molecular mechanisms, compared to the original molecule. Indeed, we observed that the immature band of ABCB4 is less pronounced after treatment with roscovitine, which was not the case with treatment at 27 °C, CsA or roscovitine analogues (Figs 1A and 2B). This would reflect the involvement of distinct molecular mechanisms between roscovitine and its structural analogues. We also observed different rescuing efficiencies of roscovitine analogues on the three distinct ABCB4 variants (compare Figs 5A,B and 6A–D) which may reflect different structural defects induced by the missense variations. It is interesting to note that the presence of a carboxyl which prevents kinase inhibition (Fig. 2A, Table 1) does not prevent rescue of ER-retained ABCB4 variants. Moreover, the presence of a cyclopentyl at position 9 and substitutions (F, Cl, CF3) seem to contribute to the ABCB4 rescuing efficacy of the analogues. Although roscovitine has been reported to partially correct CFTR-F508del function^25^, we did not observe such effect on ABCB4-I541F. These results may illustrate the difference of molecular mechanisms between the floppase activity of ABCB4 and the chloride channel function of CFTR. However, here, we provide evidence that roscovitine analogues are able to partially correct the function of ER-retained ABCB4 variants even if the mechanisms of their cell entry, passive diffusion or active uptake are not yet characterized. This opens the perspective to the use of these and related compounds as targeted pharmacotherapies for patients with ABCB4-related diseases.

The research of pharmacological therapies for ABCB4-related diseases as alternatives to liver transplantation is mainly focused on PFIC3, the most severe form of these diseases, for which the majority of homozygous or compound heterozygous patients have defects in both *ABCB4* alleles (for a review, see^41^). PC concentrations in bile of PFIC3 patients are very low due to a strongly impaired ABCB4 activity^7^. Thus, targeting patients harbouring this category of genetic variations using roscovitine analogues could be a promising therapeutic strategy in order to improve PC secretion into bile. Indeed, it has been proposed that patients with enough residual ABCB4 activity (≥7%), are better responders to UDCA therapy^42^, as this is the case for patients with milder forms of ABCB4-related diseases such as low-phospholipid associated cholelithiasis syndrome and intrahepatic cholestasis of pregnancy^41–44^. It is thus tempting to speculate that the partial rescue of ABCB4 function using roscovitine analogues would be sufficient for patients to positively respond to UDCA therapy. It is also interesting to note that UDCA treatment may contribute to the relocalisation of ABCB4 at the canalicular membrane of hepatocytes in *in vivo* systems, as proposed for ABCB11 and ABCC2 in cholestatic rat models^45,46^ and ABCB11 in PFIC2 patients^47^.

The effect of roscovitine are currently evaluated in preclinical and clinical trials in the frame of several pathologies^48^, including a recent phase 2 clinical trial in patients with cystic fibrosis (ROSCO-CF; https://clinicaltrials.gov/ct2/show/NCT02649751). The results obtained from these studies would allow to consider the repositioning of roscovitine, and more especially its more potent structural analogues, to treat ABCB4-related rare biliary diseases.

## Materials and Methods

### DNA constructs and mutagenesis

The subcloning of wild type (WT) ABCB4, isoform A (NM_000443.3), into pcDNA3 vector has been described^26^. The I541F, I490T and L556R missense ABCB4 variants were previously reported and described^9,26,49^. Site directed mutagenesis were performed using the QuikChange II XL site-directed mutagenesis kit (Agilent Technologies, Les Ulis, France), following manufacturer’s instructions, and using the primers (Eurogentec, Angers, France) described in Supplementary Table S1. The sequences of all constructs were systematically verified by automated sequencing.

### Cell culture and transfection

Human embryonic kidney (HEK-293, herein referred to as HEK; ATCC^®^-CRL-1573^TM^) cells and human hepatocellular carcinoma HepG2 (ATCC^®^- HB-8065^TM^) cells were obtained from ATCC (Manassas, VA). As we previously reported, both HEK and HepG2 cells do not express ABCB4^9,50^. Cells were grown in an incubator at 37 °C with 5% CO_2_ in Dulbecco’s Modified Eagle Medium (Gibco-Thermo Fisher Scientific, Villebon-sur-Yvette, France) containing 4.5 g/L D-glucose and supplemented with 10% heat-inactivated fetal bovine serum (Sigma, Saint-Quentin Fallavier, France), 2 mM L-glutamine, 2 mM sodium pyruvate, 100 units/mL of penicillin and 100 µg/mL streptomycin (Gibco-Thermo Fisher Scientific).

For transient transfection of HEK cells, they were seeded at subconfluent levels in the adequate tissue culture wells at least six hours before transfection. Turbofect (Thermo Fisher scientific) was used at a ratio of reagent:DNA of 2:1 according to manufacturer’s instructions. For transient transfection of HepG2 cells, subconfluent cultures were seeded in the adequate culture wells 24 hours before transfection. Lipofectamine 3000 (Thermo Fisher Scientific) was used at a ratio of reagent:DNA of 1.5:1 according to manufacturer’s instructions. Cell treatments and processing for further analyses were performed at least 16 hours post-transfection.

### Chemicals and cell treatments

Cyclosporin A (CsA) was from Santa Cruz Biotechnologies (Dallas, TX). (R)-roscovitine and its non-commercial structural analogues (Aftin-4, the metabolite M3, MRT2-163, MRT2-164, MRT2-235, MRT2-237, MRT2-239, MRT2-241, MRT2-243, MRT2-245, MRT2-248 and MRT2-249; see Fig. 2) were synthesized by ManRos Therapeutics (Roscoff, France). Each compound was solubilized in dimethylsulfoxide (DMSO) as 1000X concentrated stock solutions in order to treat cells with 5 µM to 100 µM final concentrations, using DMSO as control vehicle at the same dilution (0.1% DMSO for all conditions). The cells were treated 24 hours post-transfection with these drugs during 16 hours, except for the viability assays for which cells were treated during three days. After drug treatment, cells were used for immunoanalyses, cell viability assays or PC secretion assays.

Protein kinases and their activators or regulators (see Table 1) were expressed and purified, and their catalytic activity was assayed in the presence of a range of concentrations of each roscovitine analogue, as previously described^28,29,40^. IC_50_ values were calculated from the dose-response curves.

### Immunoanalyses

Western blots and indirect immunofluorescence were performed as previously described^9,50^. For immunoblot analyses, total cell lysates were prepared in denaturing and reducing sample buffer^51^, separated on 7.5% SDS-PAGE and transferred on nitrocellulose membranes using Trans-Blot system (Bio-Rad Laboratories, Hercules, CA). Saturated membranes were incubated with mouse monoclonal anti-ABCB4 (clone P_3_II-26; Enzo Life Sciences, Villeurbanne, France) or anti-α-tubulin (clone 1E4C11; ProteinTech, Manchester, United Kingdom) antibodies and then with peroxidase conjugated anti-mouse secondary antibodies (Sigma). Signals were detected with ECL prime western blotting detection reagent (GE Healthcare, Velizy-Villacoublay, France) and quantified in the linear range of detection using ImageJ 1.50i software (U.S. National Institutes of Health, Bethesda, MD).

For indirect immunofluorescence, HepG2 cells were grown on glass coverslips and after transfection and treatments, they were fixed and permeabilized during 1 min at −20 °C in ice-cold methanol. Then ABCB4 and ABCC2 were immunolabelled using the mouse monoclonal P_3_II-26 (IgG2b) and M_2_I-4 (IgG1) antibodies (Enzo Life Sciences), respectively. AlexaFluor^®^ 555 and AlexaFluor^®^ 488 conjugated isotype-specific secondary antibodies (Thermo Fisher Scientific) and Hoechst 33342 (Thermo Fisher Scientific) were used to label ABCB4, ABCC2 and nuclei, respectively. Images were acquired using an IX83 inverted fluorescence microscope from Olympus (Rungis, France), equipped with a UPLSAPO 60XS2 silicone immersion objective and a Hamamatsu ORCA Flash4.0 digital CMOS camera, and analysed using Olympus CellSens Dimension Desktop version 1.16 and Adobe Photoshop version 8.0.1. For each experiment, all images were acquired with constant settings (acquisition time and correction of signal intensities).

### Cell viability assays

Cell viability was assessed by the conversion of MTT (3-[4,5-dimethylthiazol-2-yl]-2,5 diphenyl tetrazolium bromide) into formazan crystals by living cells, as described^52^. In brief, HEK cells were seeded in 96-well plates in triplicate for each tested condition, including controls (no cells, no treatment, treatment with vehicle). After transient expression of ABCB4-WT as described above (to mimic the experimental conditions of the other experiments) and drug treatment during 72 hours, 125 µg/ml MTT (final concentration) was added in each well and cells were re-incubated at 37 °C during 2 hours. These conditions were optimized in order to maintain the absorbance at 540 nm of the blanks below 0.1 OD unit (negative controls) and the absorbance at 540 nm for untreated cells between 1.0 and 1.3 OD units (positive controls). Then culture media were gently washed out, cells were lysed in 100 µL of pure DMSO and absorbance at 540 nm was measured using a multiplate cytofluorimeter SpectraFluor from Tecan (Männedorf, Switzerland). Cell viability was calculated for each triplicate after background subtraction and expressed as percentage of the mean for cells treated with vehicle only.

### Measurement of ABCB4-mediated phosphatidylcholine secretion

HEK cells were seeded in 0.01% poly-L-lysine (Sigma) pre-coated 6-well plates of 10 cm² area per well (1.3 × 10^6^ cells per well). After transient transfection of the empty vector (control), plasmids encoding ABCB4-WT or its variants, cells were incubated during 24 hours at 37 °C in serum-free medium supplemented with 0.5 mM sodium taurocholate (Sigma) and 0.02% fatty acid–free bovine serum albumin (Sigma). Then, the secreted PC was quantified from the collected media using a fluoro-enzymatic assay previously described^50^. Each condition was analysed in triplicate, after background subtraction, and results were normalized to the expression levels of ABCB4 determined by immunoblots of the corresponding cell lysates.

### Statistics

Graphics and non-parametric analyses of variance tests (Kruskall-Wallis) were performed using Prism version 7.00 (GraphPad Software, La Jolla, CA). A *P* value of less than 0.05 was considered significant. If not specified in figure legends, symbols indicate the comparison between the control (or vehicle-treated) and the other tested conditions.

## Supplementary information

Vauthier - Supplementary Information

## Data Availability

The datasets generated and analysed during the current study are available from the corresponding author on reasonable request.

## References

[1] Boyer JL (2013). Bile formation and secretion. Compr Physiol.

[2] Falguières T, Aït-Slimane T, Housset C, Maurice M (2014). ABCB4: insights from pathobiology into therapy. Clin Res Hepatol Gastroenterol.

[3] Admirand WH, Small DM (1968). The physicochemical basis of cholesterol gallstone formation in man. J Clin Invest.

[4] Borst P, Zelcer N, van Helvoort A (2000). ABC transporters in lipid transport. Biochim Biophys Acta.

[5] Wang DQ, Cohen DE, Carey MC (2009). Biliary lipids and cholesterol gallstone disease. J Lipid Res.

[6] Dixon PH (2000). Heterozygous MDR3 missense mutation associated with intrahepatic cholestasis of pregnancy: evidence for a defect in protein trafficking. Hum Mol Genet.

[7] Jacquemin E (2001). The wide spectrum of multidrug resistance 3 deficiency: from neonatal cholestasis to cirrhosis of adulthood. Gastroenterology.

[8] Rosmorduc O, Hermelin B, Poupon R (2001). MDR3 gene defect in adults with symptomatic intrahepatic and gallbladder cholesterol cholelithiasis. Gastroenterology.

[9] Delaunay JL (2016). A functional classification of ABCB4 variations causing progressive familial intrahepatic cholestasis type 3. Hepatology.

[10] Vauthier V, Housset C, Falguieres T (2017). Targeted pharmacotherapies for defective ABC transporters. Biochemical pharmacology.

[11] Poupon R (2012). Ursodeoxycholic acid and bile-acid mimetics as therapeutic agents for cholestatic liver diseases: An overview of their mechanisms of action. Clin Res Hepatol Gastroenterol.

[12] Rosmorduc O, Poupon R (2007). Low phospholipid associated cholelithiasis: association with mutation in the MDR3/ABCB4 gene. Orphanet J Rare Dis.

[13] Geenes V, Williamson C (2009). Intrahepatic cholestasis of pregnancy. World J Gastroenterol.

[14] Jacquemin E (1997). Ursodeoxycholic acid therapy in pediatric patients with progressive familial intrahepatic cholestasis. Hepatology.

[15] Davit-Spraul A, Gonzales E, Baussan C, Jacquemin E (2009). Progressive familial intrahepatic cholestasis. Orphanet journal of rare diseases.

[16] Delaunay JL (2017). Functional defect of variants in the adenosine triphosphate-binding sites of ABCB4 and their rescue by the cystic fibrosis transmembrane conductance regulator potentiator, ivacaftor (VX-770). Hepatology.

[17] Gautherot J (2012). Effects of Cellular, chemical and pharmacological chaperones on the rescue of a trafficking-defective mutant of the ATP-binding cassette transporters ABCB1/ABCB4. J Biol Chem.

[18] Andress EJ (2014). A molecular mechanistic explanation for the spectrum of cholestatic disease caused by the S320F variant of ABCB4. Hepatology.

[19] Park HJ (2016). Functional characterization of ABCB4 mutations found in progressive familial intrahepatic cholestasis type 3. Sci Rep.

[20] Foxwell BM, Mackie A, Ling V, Ryffel B (1989). Identification of the multidrug resistance-related P-glycoprotein as a cyclosporine binding protein. Mol Pharmacol.

[21] Tamai I, Safa AR (1990). Competitive interaction of cyclosporins with the Vinca alkaloid-binding site of P-glycoprotein in multidrug-resistant cells. J Biol Chem.

[22] Meijer L (1997). Biochemical and cellular effects of roscovitine, a potent and selective inhibitor of the cyclin-dependent kinases cdc2, cdk2 and cdk5. European journal of biochemistry.

[23] Meijer L, Raymond E (2003). Roscovitine and other purines as kinase inhibitors. From starfish oocytes to clinical trials. Accounts of chemical research.

[24] Meijer L (2016). Modulating Innate and Adaptive Immunity by (R)-Roscovitine: Potential Therapeutic Opportunity in Cystic Fibrosis. Journal of innate immunity.

[25] Norez C (2014). Roscovitine is a proteostasis regulator that corrects the trafficking defect of F508del-CFTR by a CDK-independent mechanism. British journal of pharmacology.

[26] Delaunay JL (2009). A missense mutation in ABCB4 gene involved in progressive familial intrahepatic cholestasis type 3 leads to a folding defect that can be rescued by low temperature. Hepatology.

[27] Sormunen R, Eskelinen S, Lehto VP (1993). Bile canaliculus formation in cultured HEPG2 cells. Laboratory investigation; a journal of technical methods and pathology.

[28] Tang L (2005). Crystal structure of pyridoxal kinase in complex with roscovitine and derivatives. J Biol Chem.

[29] Bettayeb K (2012). Small-molecule inducers of Abeta-42 peptide production share a common mechanism of action. Faseb j.

[30] Tougeron D, Fotsing G, Barbu V, Beauchant M (2012). ABCB4/MDR3 gene mutations and Cholangiocarcinomas. J Hepatol.

[31] Jacquemin E (2012). Progressive familial intrahepatic cholestasis. Clin Res Hepatol Gastroenterol.

[32] Mehl A, Bohorquez H, Serrano MS, Galliano G, Reichman TW (2016). Liver transplantation and the management of progressive familial intrahepatic cholestasis in children. World J Transplant.

[33] van Wessel D (2018). The natural course of FIC1 deficiency and BSEP deficiency: Initial results from the NAPPED-consortium (Natural course and Prognosis of PFIC and Effect of biliary Diversion) [ABSTRACT]. Journal of Hepatology.

[34] Naoi S (2014). Improved liver function and relieved pruritus after 4-phenylbutyrate therapy in a patient with progressive familial intrahepatic cholestasis type 2. J Pediatr.

[35] Gonzales E (2015). Targeted pharmacotherapy in progressive familial intrahepatic cholestasis type 2: Evidence for improvement of cholestasis with 4-phenylbutyrate. Hepatology.

[36] Gordo-Gilart R, Andueza S, Hierro L, Jara P, Alvarez L (2016). Functional Rescue of Trafficking-Impaired ABCB4 Mutants by Chemical Chaperones. PLoS One.

[37] Shneider BL, Vockley J (2015). Possible Phenylacetate Hepatotoxicity During 4-Phenylbutyrate Therapy of Byler Disease. J Pediatr Gastroenterol Nutr.

[38] Pena-Quintana L, Llarena M, Reyes-Suarez D, Aldamiz-Echevarria L (2017). Profile of sodium phenylbutyrate granules for the treatment of urea-cycle disorders: patient perspectives. Patient preference and adherence.

[39] De Azevedo WF (1997). Inhibition of cyclin-dependent kinases by purine analogues: crystal structure of human cdk2 complexed with roscovitine. European journal of biochemistry.

[40] Bach S (2005). Roscovitine targets, protein kinases and pyridoxal kinase. J Biol Chem.

[41] Reichert MC, Lammert F (2018). ABCB4 Gene Aberrations in Human Liver Disease: An Evolving Spectrum. Seminars in liver disease.

[42] Davit-Spraul A, Gonzales E, Baussan C, Jacquemin E (2010). The spectrum of liver diseases related to ABCB4 gene mutations: pathophysiology and clinical aspects. Semin Liver Dis.

[43] Poupon R (2013). Genotype-phenotype relationships in the low-phospholipid associated cholelithiasis syndrome. A study of 156 consecutive patients. Hepatology.

[44] Bacq Y (2017). Ursodeoxycholic acid therapy in intrahepatic cholestasis of pregnancy: Results in real-world conditions and factors predictive of response to treatment. Dig Liver Dis.

[45] Beuers U (2001). Tauroursodeoxycholic acid inserts the apical conjugate export pump, Mrp2, into canalicular membranes and stimulates organic anion secretion by protein kinase C-dependent mechanisms in cholestatic rat liver. Hepatology.

[46] Dombrowski F, Stieger B, Beuers U (2006). Tauroursodeoxycholic acid inserts the bile salt export pump into canalicular membranes of cholestatic rat liver. Lab Invest.

[47] Varma S (2015). Retargeting of bile salt export pump and favorable outcome in children with progressive familial intrahepatic cholestasis type 2. Hepatology.

[48] Cicenas J (2015). Roscovitine in cancer and other diseases. Annals of translational medicine.

[49] Andress EJ, Nicolaou M, McGeoghan F, Linton KJ (2017). ABCB4 missense mutations D243A, K435T, G535D, I490T, R545C, and S978P significantly impair the lipid floppase and likely predispose to secondary pathologies in the human population. Cell Mol Life Sci.

[50] Gautherot J (2014). Phosphorylation of ABCB4 impacts its function: Insights from disease-causing mutations. Hepatology.

[51] Laemmli UK (1970). Cleavage of structural proteins during the assembly of the head of bacteriophage T4. Nature.

[52] van Meerloo J, Kaspers GJ, Cloos J (2011). Cell sensitivity assays: the MTT assay. Methods Mol Biol.

