# Supplementary material for: Structural analogues of roscovitine rescue the intracellular traffic and the function of ER-retained ABCB4 variants in cell models
Source: Sci Rep. 2019 Apr 30;9:6653. doi: 10.1038/s41598-019-43111-y (PMC6491434; doi:10.1038/s41598-019-43111-y)
Supplement: Supplementary file 1 — Vauthier - Supplementary Information [file 41598_2019_43111_MOESM1_ESM.pdf]

SUPPLEMENTARY INFORMATION

Structural analogues of roscovitine rescue the intracellular traffic and the function of ER-retained ABCB4 variants in cell models

Virginie Vauthier[1], Amel Ben Saad[1], Jonathan Elie[2], Nassima Oumata[2], Anne-Marie Durand-Schneider[1], Alix Bruneau[1], Jean-Louis Delaunay[1], Chantal Housset[1,3], Tounsia Aït-Slimane[1], Laurent Meijer[2], Thomas Falguières[1,*]

[1] Inserm, Sorbonne Université, Centre de Recherche Saint-Antoine (CRSA), UMR_S 938, Institute of Cardiometabolism and Nutrition (ICAN), F-75012 Paris, France.

[2] ManRos Therapeutics, Hôtel de Recherche, Centre de Perharidy, F-29680 Roscoff, France.

[3] Assistance Publique - Hôpitaux de Paris, Hôpital Saint-Antoine, Centre de Référence des Maladies Rares - Maladies Inflammatoires des Voies Biliaires & Service d'Hépatologie, F-75012 Paris, France.

[*] **Corresponding author:** Thomas Falguières, PhD – Saint-Antoine Research Center, UMR_S 938 Inserm / Sorbonne Université – 27, rue Chaligny – F-75571 Paris cedex 12, France. Phone : +33-(0)1-40-01-13-69. Fax: +33-(0)1-40-01-14-32. e-mail: thomas.falguieres@inserm.fr

Supporting Table S1. Primers used for *ABCB4* mutagenesis

Variants		Sequence(5'>3')
I490T	Sense	GTTTTCCACCACAATTGCTGAAAATACTTGTTATGGCCGTG
	antisense	CACGGCCATAACAAGTATTTTCAGCAATTGTGGTGGAAAAC
I541F	sense	GGTGGGCAGAAGCAGAGGTTCGCCATTGCACGTGCC
	antisense	GGCACGTGCAATGGCGAACCTCTGCTTCTGCCCACC
L556R	sense	CAAGATCCTTCTGCGGGATGAGGCCAC
	antisense	GTGGCCTCATCCCGCAGAAGGATCTTG

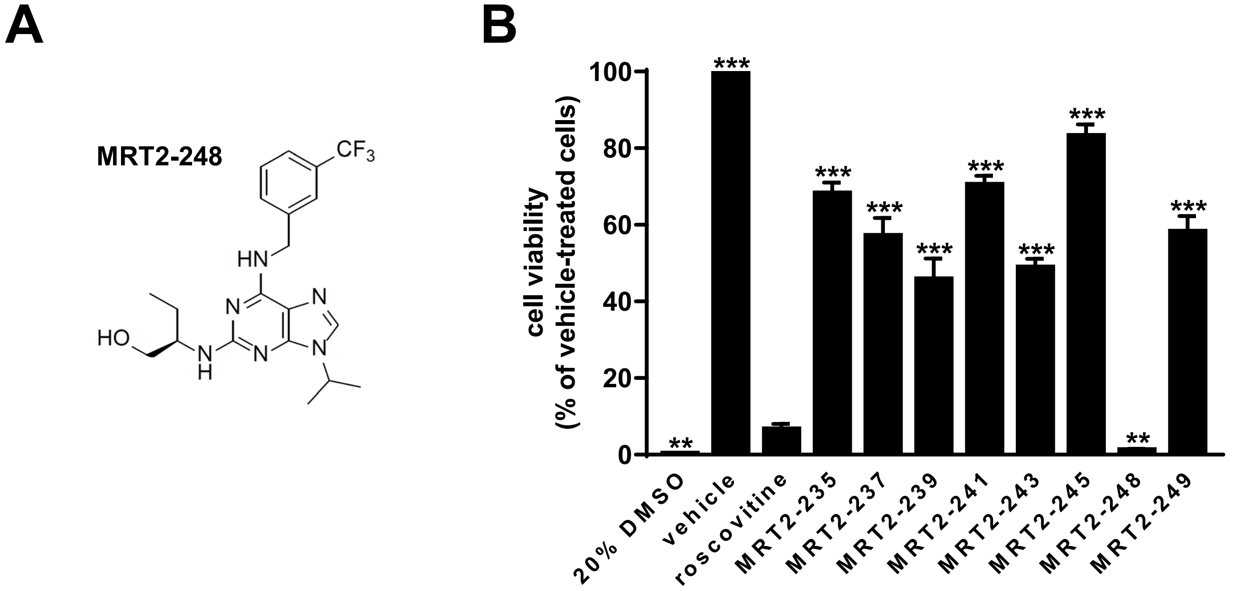

C

Kinases	CDK2/A	CDK5/p25	CDK9/T	CK1	CLK1	DYRK1A	GSK3
Roscovitine	0.080	0.210	0.533	4.3	2.9	3.33	>10
MRT2-248	0.110	0.190	0.433	0.470	1.5	2.0	>10

Refer to Table 1 for details.

Fig. S1. Cell viability assays after treatment with roscovitine analogues. A. Structure of MRT2-248. Note that this analogue is the non-carboxylated version of MRT2-249 (see Fig. 2A). **B.** After transient expression of ABCB4-WT, HEK cells were treated with 100µM of the indicated molecules, the vehicle (DMSO) or 20% DMSO (positive control of cell death) during three days. Then, cell viability was assessed by MTT assay and expressed as a percentage of the mean for vehicle-treated cells. Means (± SEM) of four independent experiments performed in triplicate are shown. Statistics indicate the comparison between the roscovitine treatment and the other conditions: $**P < 0.01$; $***P < 0.001$. **C.** IC_{50} of protein kinases after treatment with MRT2-248, as shown in Table 1 for other roscovitine analogues. Values for roscovitine are shown for comparison. Note that treatment with non-carboxylated molecules, *i.e.* roscovitine and MRT2-248, are strong CDK inhibitors and lead to massive cell death.

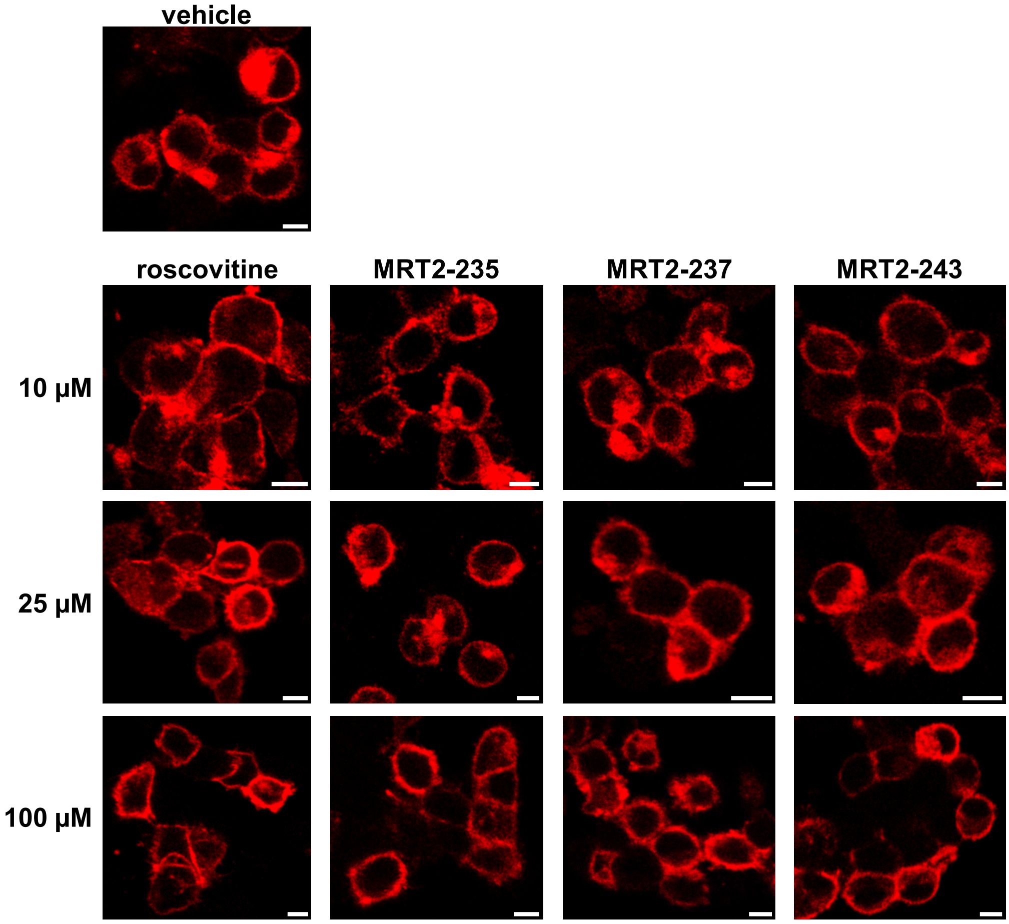

Fig. S2. Roscovitine and its analogues do not modify plasma membrane localisation of ABCB4-WT in HEK cells. After transient expression of ABCB4-WT, HEK cells were treated with the indicated concentrations of roscovitine, MRT2-235, MRT2-237 or MRT-243 as in Fig. 4B. After fixation and permeabilization of the cells, localization of ABCB4-WT (red) was assessed by indirect immunofluorescence and confocal microscopy. This figure is representative of three independent experiments. Bars: 5 µm.

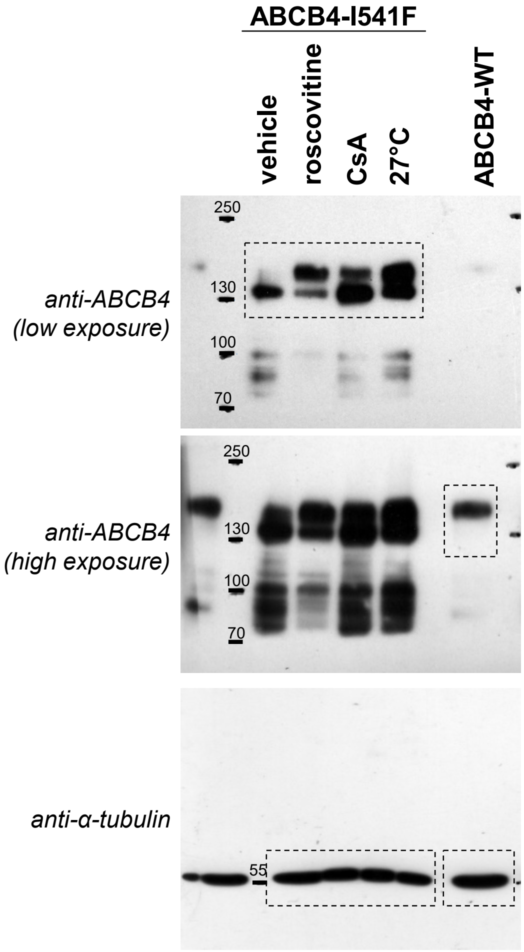

Fig. S3. Full immunoblots related to Figure 1A. These immunoblots are representative of six independent experiments. Results shown in Fig.1A are delineated by dotted rectangles. MW (in kDa) are indicated.

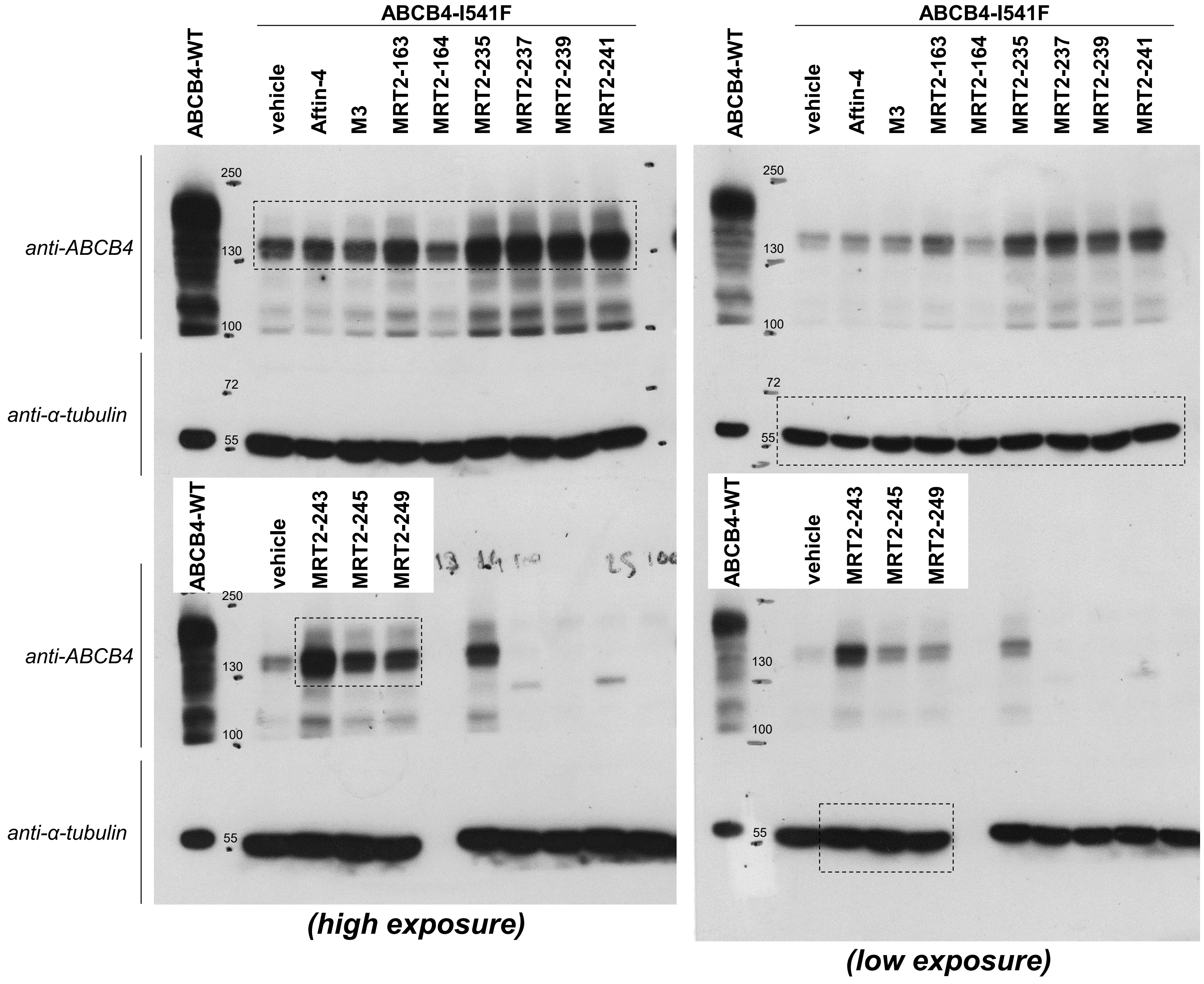

Fig. S4. Full immunoblots related to Figure 2B. These immunoblots are representative of five independent experiments. Results shown in Fig. 2B are delineated by dotted rectangles. MW (in kDa) are indicated.

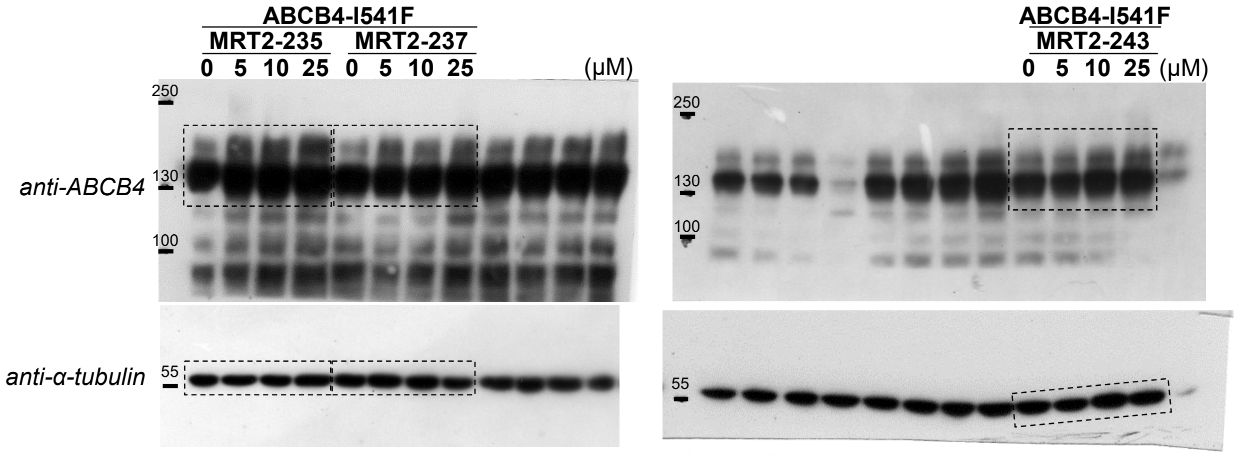

Fig. S5. Full immunoblots related to Figure 5A. These immunoblots are representative of at least five independent experiments for each condition. Results shown in Fig. 5A are delineated by dotted rectangles. MW (in kDa) are indicated.

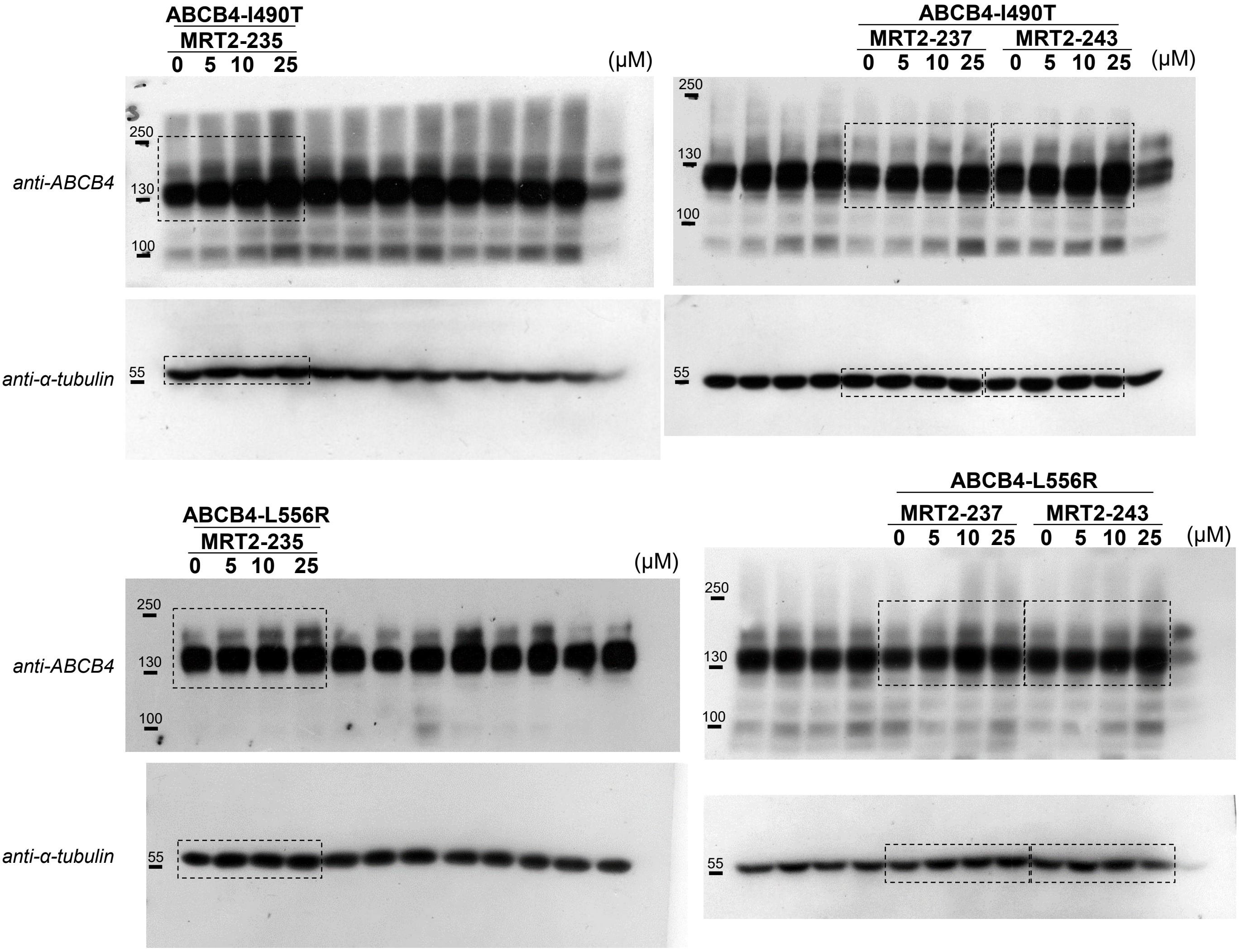

Fig. S6. Full immunoblots related to Figure 6A-B. These immunoblots are representative of at least four independent experiments for each condition. Results shown in Fig. 6A-B are delineated by dotted rectangles. MW (in kDa) are indicated.
